# Assessing the Gaps in Medical Student Usages of Professional Interpreter Services When Caring for Patients With Limited English Proficiency

**DOI:** 10.7759/cureus.100759

**Published:** 2026-01-04

**Authors:** Antonia Oladipo, Claire Wolford, Christina Sheedy, Ofelia Martinez, Sara Bittman

**Affiliations:** 1 Obstetrics and Gynecology, Hackensack University Medical Center, Hackensack, USA; 2 Family Medicine, CentraState Medical Center, Freehold, USA; 3 Medical Education, Hackensack University Medical Center, Hackensack, USA

**Keywords:** interpreter use, medical education, medical interpreter, non-english speakers, professional medical interpreter

## Abstract

Aim: Patients with limited English proficiency (LEP) have poorer access to healthcare, and their interactions with the healthcare system are often truncated. This qualitative-thematic study assesses undergraduate medical students to determine whether students observed disparities in the treatment of LEP patients both by themselves and by other members of the healthcare team.

Materials and methods: A cross-sectional survey was delivered to all undergraduate medical students at a single United States allopathic medical school to assess student comfort with interpretation modalities, perceptions of their own behavior, and perceived behaviors in other members of the healthcare team.

Results: Undergraduate medical students across all cohorts (n=60, M1=20.0%, M2=26.7%, M3=26.7%, M4=26.7%) observed that LEP patients spent less time with providers (61.7%), received less time spent discussing a diagnosis (60.0%), and in many cases, no translation services were used during the encounter (60.0%). The most common challenge identified by medical students was accounting for the additional time necessary to communicate across a language barrier (30.0%). The most common solutions recommended included increasing the number of translation devices and access to information for medical students at each clerkship site.

Conclusion: While the response rate was low (n=60, 12.2%), this survey yields preliminary findings indicating that undergraduate medical students can provide insight into specific behaviors and attitudes found in the treatment of LEP patients. A future longitudinal study of undergraduate medical students is recommended to assess if comfort with translation and favorable attitudes towards working with LEP patients improve over time.

## Introduction

Evidence suggests that patients with limited English proficiency (LEP) have poorer access to healthcare and consequently experience worse outcomes [1,2]. In a pediatric emergency room setting, 58% of English-speaking parents followed up with a physician, while only 40% of LEP patient parents did so [3]. In an adult emergency room setting, a study by Waxman et al. identified that patients with LEP are more likely to receive additional diagnostic testing when presenting with abdominal pain, including three times the rate of abdominal computed tomography scans [4]. Additionally, LEP patients without interpretive services were more likely to be readmitted [5]. Similarly, a study assessing English language proficiency and stroke risk found that LEP patients with atrial fibrillation were less likely to be taking warfarin compared to English-speaking patients [6]. Additionally, even when health and lifestyle factors such as smoking and alcohol use, hypertension, and lipid disorder were accounted for, LEP patients were more likely to have a history of diabetes (41.4%) than English-speaking patients (10.2%), potentially indicating that these patients were not adequately identified and managed in the pre-diabetes stage [6]. These findings also extend to genetic counseling, exemplified by lower informed consent scores for consultations with Latinx patients across a language barrier in eight prenatal genetics clinics [7]. Furthermore, a 2022 systematic review of articles assessing surgery outcomes in LEP patients identified poorer postoperative pain control and limited understanding of discharge instructions when compared with English-speaking patients [8].

The use of professional interpretive services can help lessen healthcare disparities between English-speaking and LEP patients. A 2007 review by Bennett et al. of outpatient clinics, emergency departments, and inpatient settings studied patients’ access to provider language across three categories: professional interpreters, ad hoc interpreters, and no interpreters [9]. Ad hoc interpreters were defined as bilingual family, friends, staff members, or third parties with no formal training in medical translation [9]. This review found that clinical error rates were lower when professional interpretive services were used with LEP patients (53%) when compared to ad hoc interpreters (77%) [9]. Additionally, patients with interpreters had lower rates of return to the emergency department [9]. These error rates are also seen in an acute care setting, such as an emergency room. In a pediatric emergency department, a study of 13 encounters yielded 396 interpreter errors, with a mean of 31 per encounter [10]. 63% of these errors had potential clinical consequences, and errors committed by ad hoc interpreters were significantly more likely to have potential clinical consequences (77%) than hospital interpreters (53%). Other systematic reviews identified that professional interpreters appeared to raise the quality of clinical care for LEP patients to approach or equal that of English-speaking patients [11].

Considering how effective professional interpreters are, it is concerning that physician use is not ubiquitous. Wilson et al. in 2021 describes 273,796 outpatient physicians’ interpreter use, 78.7% of respondents used “some kind” of interpreter, but 39.9% reported “never” using professional interpreters and only 29.5% of physicians “regularly” using professional interpreters [12]. Physicians reported frequent use of ad hoc interpreters, and these are associated with poorer patient outcomes [12]. Alarmingly, a qualitative study interviewing internal medicine resident physicians at two large, urban teaching hospitals found normalization of underusing professional interpretive services in favor of ad hoc interpreters, despite knowing that professional interpreters are associated with higher quality of care [13]. The residents expressed attitudes of “just getting by” as justification for interpreter non-use. Often, residents felt that they had to weigh a desire for accurate communication against other competing demands on their time [13]. These findings were consistent with those found among pediatric residents, where 80% of residents admitted to avoiding communication with LEP families, and 52.5% of residents believed that LEP families in their care “never” or “sometimes” understood their child’s diagnosis [14]. 

Because these behaviors were identified among resident physicians, undergraduate medical students are ideal candidates for LEP-specific training and intervention. There is a paucity of research assessing the medical student experience and preparedness with LEP services. A 2011 study found that self-reported skill in interpreter use, exposure to LEP patients, and year in medical training were correlated with increased self-reported preparedness to care for LEP patients [15]. Additionally, curricular adjustments to include LEP-specific trainings have shown improvements in preparedness to care for LEP patients among their student population, as evidenced at Albert Einstein College of Medicine [16]. Albert Einstein College of Medicine’s curriculum previously included a 16-session longitudinal course entitled “Patients, Doctors, and Communities” that includes four introductory sessions in the spring of the second year and then runs concurrently with clerkships in the third year of medical school. They created a session called “Cross-Cultural Communication-Using an Interpreter,” a two-hour session, which included a full hour of practice with an ad hoc interpreter that included pauses for feedback [16]. After completing the session, 77.3% of students agreed or strongly agreed that they felt more prepared to communicate with a patient who has LEP, 77.3% of students agreed or strongly agreed that they felt more prepared to give instructions to an untrained interpreter, and 76.4% of students agreed or strongly agreed that they felt more prepared to access a hospital language line [16]. A curriculum intervention at Stanford University revealed similar findings. The addition of a single standardized interpretation session with medical students, LEP patients, and interpreters improved medical student performance as assessed by session preceptors (90.9%) and medical student confidence (81.3%) [17]. Additionally, a curriculum intervention at California University of Science and Medicine found similar results, even when that intervention is delivered digitally: the addition of a Medical Interpreter website training increased the average confidence of first-year medical students to work with LEP patients. Out of a five-point scale, where 5 is high confidence and 1 is low confidence, first-year medical students’ self-assessment increased from 2.4 points ± 0.4 pre-lesson to 3.9 ± 0.3 post-lesson, reflecting an average improvement of +1.5 points [18].

While current literature does demonstrate the effectiveness of interpreter training for medical students, there is very little information regarding medical students' overall perception of how LEP patients are treated in the healthcare system. The primary aim of our study is to gather the medical student perspective, both of themselves and of the healthcare team, of how LEP patients are treated via a cross-sectional survey. Our secondary aim is to elicit any proposed solutions they may have.

This article was previously presented as an abstract at the 2023 ChangeMedED conference on September 28, 2023. 

## Materials and methods

Study design

A questionnaire-based electronic survey was distributed in 2022 via email to all medical students at Hackensack Meridian School of Medicine. Data were collected via an electronic data collection platform, REDCap (Vanderbilt University, Nashville, TN), for a one-month period prior to analysis. The questionnaire is modified from an existing cultural-competence survey designed for residents to assess comfortability with cross-cultural care across multiple dimensions [19]. Our team adapted this survey to the undergraduate medical student population and tailored our questions to matters concerning LEP patients. Responses were limited to one per participant. Three reminder emails were sent, one email per week. The survey was conducted anonymously, the research team did not communicate with participants, and no compensation was offered. Only completed surveys were included for analysis. 

The questionnaire was developed by this research team and included 18 total items, with 17 included for analysis. Four questions pertained to the demographics of the student respondent, three questions pertained to student experience with translation services in the clinical setting, five questions pertained to the undergraduate student’s perspective on using translation services, three questions pertained to the medical student’s perspective on communicating with LEP patients, two questions pertained to the medical student’s perception of other members of the healthcare team, and one question polled students on a range of potential means to improve medical student interaction with LEP patients. Question format ranged from yes-no questions, a Likert-type scale [20], and multiple-response questions. A single open-ended demographic question was excluded from our analysis due to poor generalizability. 

Study population

Four hundred ninety-one undergraduate medical students at Hackensack Meridian School of Medicine were invited to complete the survey. All undergraduate medical students were included in the study; there were no exclusion criteria. Demographics of respondent students broadly reflect that of the Hackensack Meridian School of Medicine student body and are reported. 

Ethics statement

This study was conducted with Internal Review Board (IRB) approval in 2022 in accordance with IRB protocol Pro2022-0216. Informed consent was obtained from all participants in this study. 

Analysis

Responses were read and coded into REDCap by a single coder. Descriptive analysis was conducted using Statistical Package for Social Sciences (SPSS) version 20 (IBM Corp., Armonk, NY) with α=0.05. Two additional coders conducted qualitative-thematic analysis for survey line items found in common between medical students' perceptions of themselves and other members of the healthcare team using the Braun and Clarke method [21]. One of the two coders assigned names for these common themes.

## Results

Of the eligible 491 students receiving the survey, 60 students responded (12.2%). Demographic information and educational attainment were as follows: 38 (63.3%) respondents identified as cis-female and 22 (36.7%) identified as cis-male (Table 1). 39 (65.0%) of our respondents identified as white, 19 (31.7%) of our respondents identified as Asian, and the remaining 2 (3.3%) respondents identified as Hispanic. Of note, the majority of LEP patients in the United States are Hispanic, while only a small minority of respondents to this survey share that identity (Table 2). 

**Table 1:** Sex and gender identity of medical students. Students identify their sex and gender identity from a provided list of options.

Sex	Sex of study participants (n=60)
Female	63.3% (n=38)
Male	36.7% (n=22)
Intersex	0% (n=0)
Prefer not to say	0% (n=0)
Identity	Gender identity of study participants (n=60)
Cis-female	63.3% (n=38)
Trans-female	0% (n=0)
Cis-male	36.7% (n=22)
Trans-female	0% (n=0)
Nonbinary	0% (n=0)
Prefer not to say	0% (n=0)

**Table 2:** Race of study participants. The preponderance of medical student respondents is white (65.0%) and Asian (31.7%). Hispanic respondents made up only 3.3% of the dataset.

Race	Race of study participants (n=60)
American Indian or Alaska Native, non-Hispanic	0% (n=0)
Asian, non-Hispanic	31.7% (n=19)
Black or African American, non-Hispanic	0% (n=0)
Hispanic	3.3% (n=2)
Native Hawaiian or Other Pacific Islander, non-Hispanic	0% (n=0)
White, non-Hispanic	65.0% (n=39)

Educational attainment was evenly distributed among respondents: 12 first year students, 16 second year students, 16 third year students, and 16 fourth year students (Table 3), that said, while percentages from the total respondent pool are roughly equal between the four years, it should be noted that this survey was distributed at a time when Hackensack Meridian School of Medicine was expanding their student body with each successive cohort. Additionally, a number of Hackensack students graduate in three years through the accelerated Phase 3 Residency pathway. As a result, in 2022, the M1 class had approximately 167 students, while the M4 class had 34 students, due to a smaller initial class size and a significant number of early graduates. As a result, the M4 sample is likely more representative of the attitudes of the M4 student cohort when compared to the M1 and M2 samples. 

**Table 3:** Year in medical school.

Year in medical school	Number of students (n=60)
M1	20.0% (n=12)
M2	26.7% (n=16)
M3	26.7% (n=16)
M4	26.7% (n=16)

After obtaining demographic information from survey respondents, survey items focused on medical student perceptions regarding LEP patients and interpretive services in the clinical setting (Appendix A). Survey items can be subdivided into questions regarding experience with translation assessed in a yes-no format (Table 4), perceptions of self and others' behaviors towards LEP patients assessed in a multiple response format (Tables 5, 6), confidence with translation services assessed via a Likert-type scale (Table 7), and identification of specific translation difficulties and modes of translation (Tables 8-10), student preference for interpretation modality (Table 11), and perceived helpfulness of proposed solutions all assessed via multiple response format (Table 12). A single open-response question regarding other languages spoken by medical students was disregarded from our final analysis due to a failure to differentiate fluency level and native-language status. 

**Table 4:** Yes-or-no survey questions regarding English proficiency. Yes-or-no questions were asked in the LEP survey to medical students. LEP: limited English proficiency.

Survey question	Affirmative response (yes)	Negative response (no)
Have you ever had to communicate with patients with limited English proficiency (LEP)?	98.3% (n=59)	1.7% (n=1)
Do you feel comfortable navigating the use of translation services in a clinical setting?	81.7% (n=49)	18.3% (n=11)
Did you get training on how to effectively use translator services in a clinical setting?	58.3% (n=35)	41.7% (n=25)
Do you think there is a difference in how English-speaking patients are cared for by medical students versus those with LEP?	90% (n=54)	10% (n=6)
Do you think there is a difference in how members of the healthcare team treat patients who do not speak English compared to those with LEP?	88.3% (n=53)	7 (11.7%)

Survey results demonstrated that medical students perceived LEP patients to receive worse treatment in several key areas, both by healthcare providers and by medical students. 53 (88.3%) respondents felt that healthcare providers treated patients differently based on their English proficiency (Table 5). When asked to identify areas where medical students witnessed worse treatment for LEP patients, 37 (61.7%) reported less time spent with patients, 36 (60%) respondents reported that no translation services were used, 36 (60.0%) reported less time spent discussing a diagnosis or next steps, and 18 (30.0%) reported longer wait times. Worryingly, 17 (28.3%) of respondents witnessed the dismissal of patient concerns and 13 (21.7%) of respondents reported that LEP patients received a decreased number of visits and/or follow-up. Medical students' own attitudes often mirrored the behaviors they observed in providers when working with a healthcare team. When asked if there was a difference in how English-speaking and LEP patients were cared for by themselves as medical students, 54 (90.0%) respondents said yes (Table 6). When asked about specific areas of difficulty, 42 (70.0%) students perceived seeing LEP patients as a larger time commitment, 39 (65.0%) asked fewer open-ended questions, 37 (61.7%) felt more hesitancy to see patients with LEP, and 32 (53.3%) reported asking fewer questions overall when compared to English-speaking patients. There was one notable exception to this trend: 17 medical students (28.3%) observed that healthcare providers made assumptions about a patient based upon their perceived culture, ethnicity, or race without eliciting the patient’s beliefs/perspectives, whereas only 8 (13.3%) reported that behavior in themselves. These results could be explained by positive self-belief or a real difference in attitudes.

**Table 5:** Have you witnessed any of the following by other healthcare providers in relation to patients with LEP? Select all that apply. Students indicate behaviors they witnessed from other members of the healthcare team while caring for LEP patients. LEP: limited English proficiency.

Witnessed behaviors	Number of students (n=60)
Longer wait times	30.0% (n=18)
Decreased number of visits/follow-up from provider while patient is hospitalized	21.7% (n=13)
Dismissal of patient complaints or concerns	28.3% (n=17)
Assumptions about a patient based on their presumed culture, ethnicity, or race without actually eliciting the patient’s belief/perspective	28.3% (n=17)
More readily labeled “noncompliant”	18.3% (n=11)
No use of translation services for patients with LEP	60.0% (n=36)
Less time spent interviewing patients	61.7% (n=37)
Less time explaining diagnosis or next steps	60.0% (n=36)
Less time spent answering patient questions	53.3% (n=32)
Less open-ended questions were asked	58.3% (n=35)
Different treatment/testing options offered to patients with LEP compared to those who do	6.7% (n=4)
None of the above	16.7% (n=10)

**Table 6:** Have you experienced difficulty with any of the following with respect to patients with LEP? Select all that apply. Self-identified difficulties medical students experienced while working with LEP patients. LEP: limited English proficiency.

Witnessed behaviors	Number of students (n=60)
Felt more hesitancy to see patients with LEP	61.7% (n=37)
Perceived seeing patients with LEP as a bigger time commitment	70.0% (n=42)
Decreased wiliness to revisit/follow up on a patient due to a need for obtaining translation	28.3% (n=17)
Asking less questions when gathering a history than you would for a patient that does speak English	53.3% (n=32)
Asking less open-ended questions than you would with a patient that does speak English	65.0% (n=39)
Made assumptions about a patient based on their presumed culture, ethnicity, or race without eliciting the patient’s beliefs/perspectives	13.3% (n=8)
None of the above	8.3% (n=5)

**Figure 1:**
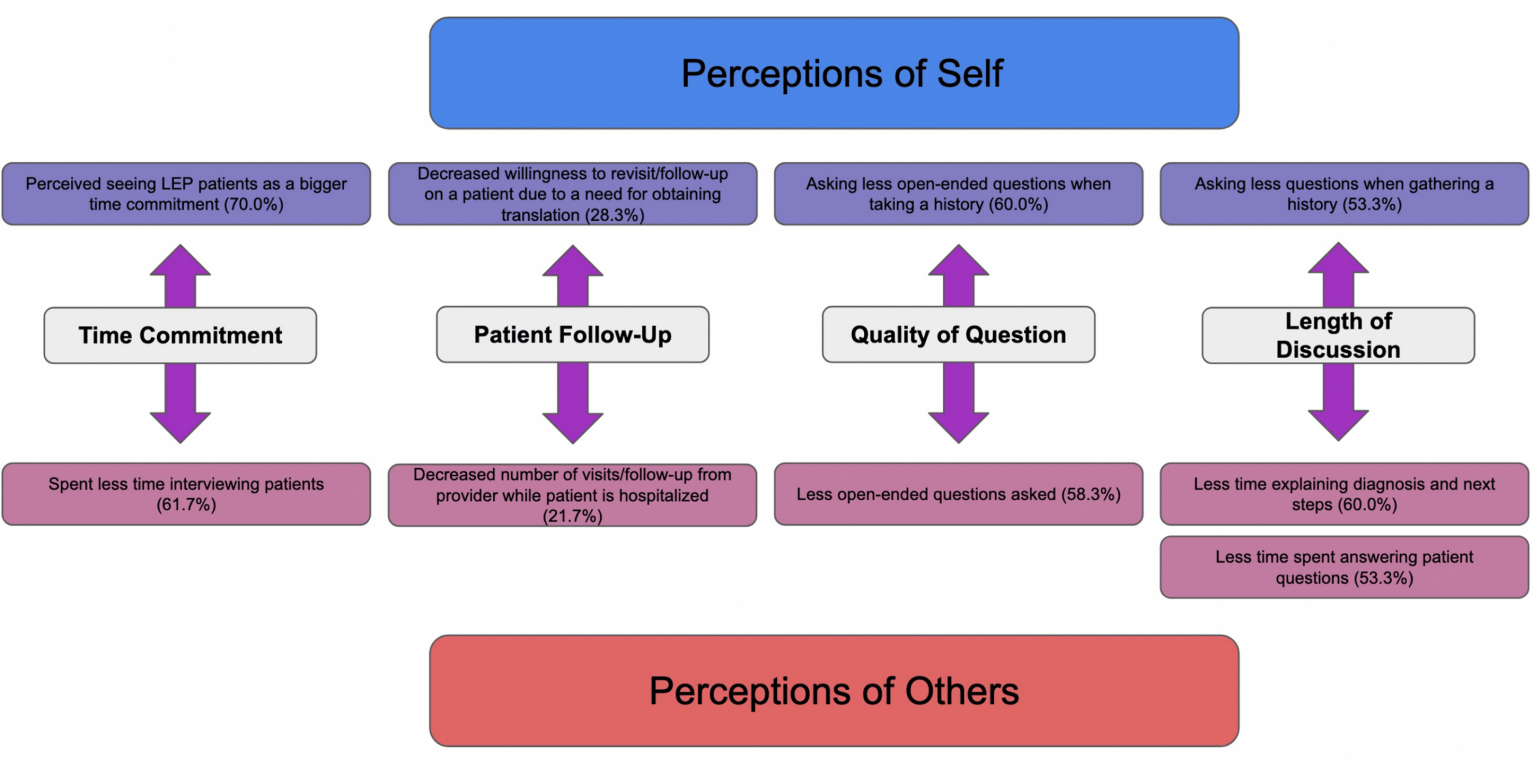
Comparison of medical student perceptions of personal and others' behavior. Illustration of comparable behaviors between medical students' perceptions of their own interactions with LEP patients and those of other members of the healthcare team, taken from Tables 5, 6. The percent of student assent associated with each finding is reported alongside its respective bubble. Behavior categories such as "time commitment," "patient follow-up," etc., demonstrate the similarity between the datasets. LEP: limited English proficiency.

Figure 1 illustrates the significant overlap between behaviors that medical students perceived in themselves and in other members of the healthcare team. Of note, while an overwhelming majority of medical students (70.0%) believed seeing LEP patients to be a greater time commitment, when observing other members of the healthcare team, they perceived a shorter total interview time with this patient population (61.7%). If both of these self-reports are accurate, this suggests that the rate-limiting step in interviewing an LEP patient concerns the acquisition and set-up of interpretation technology, rather than translating clinical information. 

Table 7 details student self-assessment of their training with translation services, with most students reporting a moderate level of training. Currently at Hackensack Meridian School of Medicine, students are trained to use a phone service called the Language Line during their preclinical years as part of the Human Dimension course, a longitudinal class focusing on social issues in medicine. The language line is also used in certain standardized patient encounters in the clinical years. However, students are not formally trained on all mediums of translation, including video and in-person interpretation, both of which are provided at various clinical sites in the Hackensack Meridian Health network.

**Table 7:** How would you rate your training with translation services? Results from a Likert-type scale assessing student self-ratings on their training with translation services, alongside the associated frequency distribution. The majority of respondents felt that they were moderately trained on translation services, with a majority of self-ratings at 2, 3, and 4.

Total count (N)	Min (poor)	Max (excellent)	Mean	Standard deviation	Frequency distribution				
					1	2	3	4	5
60	1	5	3.07	0.970	2	17	19	19	3

When asked to identify challenges to LEP patient care, 37 (61.7%) students reported issues accounting for time medical students 36 (60.0%) reported issues forming connections, and 34 (56.7%) reported issues with phrasing questions/concepts. When asked about the specific difficulties medical students have experienced while accessing translation services, 36 (60%) had difficulty locating in-person interpreters, 36 (60.0%) had difficulty finding available iPads for video translation, 31 (51.7%) had difficulty accessing the translator line via telephone, 28 (46.7%) had difficulty connecting with an interpreter via phone or video in a timely manner, and 22 (36.7%) had difficulty accessing the translator line via video (Tables 8, 9). These issues are easily solvable but can be time-intensive. Additional obstacles included low volume on phone/video affecting communication (n=32, 53.3%) and issues with asking open-ended questions.

**Table 8:** Have you experienced difficulty with any of the following? Select all that apply. Students indicate areas of difficulty they experienced when communicating with LEP patients. LEP: limited English proficiency.

Translation difficulty	Number of students (n=60)
Accessing translator line via telephone	51.7% (n=31)
Accessing translator line via video	36.7% (n=22)
Finding available iPads for video translation	60.0% (n=36)
Finding available in-person interpreters	60.0% (n=36)
Connecting with an interpreter via phone or video within a timely manner	46.7% (n=28)
Need for an interpreter for a rare dialect or language that was not available	40.0% (n=24)
Poor internet connection affecting use of translation services	36.7% (n=22)
Low volume on phone or video affecting ability to communicate with the patient	53.3% (n=32)
Asking open-ended questions while using translation services	55.0% (n=33)
Speaking in short sentences while using translation services	55.0% (n=33)
Concern for inaccurate translation	66.7% (n=40)
None of the above	5.0% (n=3)

**Table 9:** What do you find to be the most challenging part of communicating with a patient with LEP? Select all that apply. Students indicate personal challenges while communicating with LEP patients. LEP: limited English proficiency.

Translation difficulty	Number of students (n=60)
Accessing translation services via video	25.0% (n=15)
Accessing translation services via phone	25.0% (n=15)
Accessing translation services via in-person interpreter	31.7% (n=19)
Phrasing questions in a way that is easy to be translated	56.7% (n=34)
Accounting to possible increased time spent with patient due to need for translation use	61.7% (n=37)
Forming a connection with a patient while using translation services	60.0% (n=36)
Expressing empathy to the patient	35.0% (n=21)
Conducting a physical exam while using interpreter	21.7% (n=13)
Other	8.3% (n=5)

Our study briefly assessed medical student use of and preference for translation medium from the following options: phone, video, in-person professional interpreter, patient’s family member, or proficient healthcare team member. The most commonly used method of interpretation was a patient's family member, with 83.3% of medical students reporting having used a family member for interpretation (Table 10). However, 0% of medical students preferred family member interpretation; the most commonly preferred mode of interpretation was an in-person professional interpreter at 78.3% (Table 11).

**Table 10:** Select all translation methods you have used with patients. Students indicate which methods they used to communicate with LEP patients. LEP: limited English proficiency.

Translation method	Number of students (n=60)
Interpreter via video	63.3% (n=38)
Interpreter via phone	75.0% (n=45)
In-person interpreter	33.3% (n=20)
Patient’s family member as interpreter	83.3% (n=50)
Other healthcare worker as interpreter	81.7% (n=49)

**Table 11:** If given the choice, what translation method would you prefer to use? Students indicate a single preferred method for communication with LEP patients. LEP: limited English proficiency.

Translation method	Number of students (n=60)
Interpreter via video	15.0% (n=9)
Interpreter via phone	5.0% (n=3)
In-person interpreter	78.3% (n=47)
Patient’s family member as interpreter	0% (n=0)
Other healthcare worker as interpreter	1.7% (n=1)

In addition to assessing medical students' perceptions of current interactions with LEP patients, we also asked medical students if they felt certain targeted interventions would be beneficial in the treatment of LEP patients in the future (Table 12). The most favored solutions by medical students were: increasing access to translation services (n=48, 80.0%), providing medical students with more information about which translation services are available at each clinical site (n=43, 71.7%), and decreased wait times for available translators (n=32, 53.3%). Additionally, students felt positively about solutions that increased LEP-specific trainings for medical students: 34 (56.7%) students supported training on how to use translation services specifically in the clinical setting, 28 (46.7%) supported the creation of an additional refresher training on using translation services directly before beginning clerkships, and 26 (43.3%) supported improved training on how to use translation services effectively. Interestingly, even though time constraints were identified as a major factor in the perceived differential treatment of LEP patients, only 17 (28.3%) medical student respondents supported the addition of a timer added to the electronic medical record system to indicate how long patients with LEP have been waiting to be seen by a provider.

**Table 12:** Do you think any of the following would improve medical student interactions with patients with LEP? Select all that apply. Students indicate which proposed solutions they believe will be helpful in increasing access to care for patients with LEP. LEP: limited English proficiency.

Proposed solution	Number of students (n=60)
Improved training on how to use translation services effectively	43.3% (n=26)
Refresher training on the use of translation services directly before beginning clerkships	46.7% (n=28)
Training on how to use translation services, specifically in a clinical setting	56.7% (n=34)
More information provided on what translation services are available at each clinical site	71.7% (n=43)
Improved identification of patients with LEP in Epic	40.0% (n=24)
Timer added to Epic to indicate how long patients with LEP have been waiting to be seen by a provider	28.3% (n=17)
Increased access to translation services (i.e., more iPads available)	80.0% (n=48)
Decreased wait times for available translators (i.e., more translators available)	53.3% (n=32)
Other	8.3% (n=5)

## Discussion

**Figure 2:**
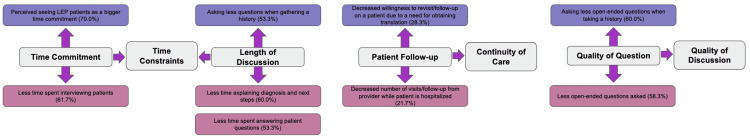
Thematic organization of medical student perceptions of personal and others' behavior. Thematic organization of medical student perceptions of their own behavior and the behavior of others on the healthcare team. Line items from Figure 1 in red and blue have been reorganized into larger themes that reflect known disparities in LEP patient care. LEP: limited English proficiency.

Our study found that medical student respondents perceived differences in their own behavior towards LEP patients that paralleled differences they perceived in other members of the healthcare team. For example, 70.0% of medical students perceived “seeing LEP patients as a bigger time commitment,” and 61.7% of medical students perceived other members in the healthcare team as “spending less time interviewing patients.” The comparable themes found in both self and others’ behavior can be identified using Braun and Clarke's model for qualitative theme assessment [21]. Figure 1 above depicts these data points with initial codes. Figure 2 below organizes these codes into three major themes: time constraints, continuity of care, and quality of discussion. Each theme identifies a perceived barrier to LEP patient care found in common between students and other members of the healthcare team.

Time constraints are consistently identified as the largest barrier to delivering appropriate care to LEP patients, both in perceived time commitment and in the length of the discussion itself. Our student perceptions align with prior data; time constraints are consistently identified as a barrier to LEP patient care in both residency programs and private practice settings, in addition to the findings at our clinical sites. For example, a study of outpatient physicians found that for clinicians who had not used an interpreter for a given appointment but wished they had, 79% reported that interpreter non-use resulted from time constraints [22]. Furthermore, a study of pediatric residents in an academic medicine setting found that 75% residents used hospital interpreters “never” or “sometimes”, and 52% of residents relied on proficient colleagues instead [14]. When interpreter non-users were asked about their non-use, time was one of the most common responses [14]. 

Interestingly, despite a larger perceived commitment to see an LEP patient, medical students in our study also perceived that less time was actually spent in the interview. This shortened interview time could result from over-compensation. Alternatively, the overall encounter time could be longer due to a combination of accessing the translation device, setting up the translation device, gathering information about the interpreter for documentation purposes, and the interview itself. As a result, lower time spent in the interview portion could be an attempt to gain back lost time. Due to the presence of additional steps like acquiring the translation service, it is logical that encounters with LEP patients would take at least a little more time. However, the rate-limiting step of LEP patient encounters is as yet unknown, both in our clinical sites and in the literature more broadly. A potential future research direction could include recording LEP and English language encounters and timing various stages of the appointment to determine where the most time is lost. Further studies could attempt interventions targeted to whichever stage of the appointment is determined to be rate-limiting. 

Our study also found that medical students perceived lower continuity of care with LEP patients. Medical students perceived that other members of the healthcare team were less likely to follow up with LEP patients, and they observed within themselves a lower willingness to do so. Regarding other members of the healthcare team, these perceptions by medical students do, unfortunately, align with prior data. As described above, one pediatric emergency room identified that only 40% of LEP patients received follow-up care after discharge, compared with 58% of English-speaking patients [3]. Additionally, O’Leary et al. identified that 80% of pediatric emergency room residents at a single-institution program admitted to avoiding communication with LEP families [14]. 

Regarding medical students’ perceptions of their own behavior, the phrasing of the survey line item “willingness” (Table 6) reveals an internal resistance to see LEP patients when compared to English-speaking patients. According to the theory of planned behavior (TBP), willingness to perform an action consists of three separate components: attitudes towards the behavior, perceived behavioral control (perceived ease of task), and subjective norms around the behavior [23]. Determining the root of this decreased willingness to follow up with LEP patients is crucial for targeted intervention, and further study is needed to make any conclusions. A longitudinal survey could assess which component of TBP has the greatest influence on student willingness to follow up. If student willingness increased over time, and they indicate on survey questions that their reluctance is due to difficulty accessing materials, low confidence when speaking with a translator, etc., we could conclude that perceived behavioral control is a major determinant of student attitudes towards LEP patients. However, if student willingness decreased over time, and quantitative measures demonstrate that their attending physicians spend less time with LEP patients, we could argue that subjective norms like implicit bias or mirroring attending behaviors may be at fault. Without objective measures like minutes spent with LEP patients and student attitudes over time, any inferences about student willingness are purely speculative.

The phrasing of this survey line item, “willingness,” reveals internal beliefs about the medical student that could be targeted for change in the future, thereby increasing follow-up rates when these students become residents. However, determining the motive for this reluctance is crucial. Further study in this area is needed to make any definitive conclusions. Specifically, a cross-sectional survey on student attitudes around follow-ups with patients focusing on length of interaction, comfortability with patient-centered communication with a third party involved, and potential difficulty with perspective-taking cross-culturally would be useful. Additionally, a longitudinal study could be performed across students’ clerkship year, to see if decreased willingness to follow-up with LEP patients lessened or intensified as students gained more experience with interpreted patient encounters. If students were more willing to follow-up over time, inexperience may be the likely culprit. However, if these attitudes intensified, students could be mirroring behavior seen in their attendings for increased social desirability.

Our last common theme found in both medical student perceptions of themselves and in others was a change in the quality of discussion with LEP patients. Students perceived that both they and other members of the healthcare team asked fewer open-ended questions when compared to encounters with English-speaking patients. While our study is limited by self-report, if students and other team members are in fact asking fewer open-ended questions, they may be failing to elicit comprehensive information from patients. A study of 1,200 medical student interviews found that the use of open-ended questions like “What brings you here?” and “What is your main question or concern for today?” yields a higher total amount of information obtained from patients during an interview (F=41.0, p<0.0001) [24]. Unfortunately, there are no quantitative studies of this nature in the setting of interpreted patient encounters. This makes our finding potentially useful, but also difficult to contextualize within the medical literature. We would recommend a future quantitative study to determine the percentage of open-ended vs closed-ended questions in an interpreted encounter when compared to an English-speaking encounter (with corresponding volume of information obtained) in order to assess whether LEP patients could be better served by altering question style. If significant, further studies could be designed to assess the impact of interpreter trainings that focus on question style with a goal of improving outcomes. 

While this was not identified as a major theme in medical student perceptions of others’ behavior, it should be noted that medical students did view it as difficult for themselves to form “a connection with a patient while using translation services” (Table 9). Difficulty in forming connections could have many possible sources. Potentially, students could struggle with cultural competency, and this is emerging in patients without a shared language. Alternatively, students could simply be uncomfortable forming a connection with a third party, the interpreter, in the conversation. Further study, including a cultural competence assessment like the Inventory for Assessing the Process of Cultural Competence among Healthcare Professionals-Student Version (IAPCC-SV), a validated cultural competence scale based on the Campinha-Bacote model designed for students, could help inform the origin of this difficulty [25].

In addition to student perceptions of self and others’ behavior, this study also assessed which mode of interpretation medical students used with LEP patients. 83.3% of medical students used a family member for translation, despite 0% of medical students preferring this mode of interpretation (Table 11). This apparent contradiction could be explained by a lack of access to alternative translation options; family members could be used when no other mode of interpretation is available or expedient. When contextualized by our findings in Tables 5, 6 regarding (perceived or real) time constraints to interview LEP patients, this discordance between real and preferred methods of translation may be a result of students' need to perform quickly. A review of the literature indicates that the most common reason for using ad hoc interpreters is a lack of expedient options. In a study of 39 healthcare professionals from five specialties, in-depth interviews revealed that time constraints were the largest driver towards using ad hoc interpretation [26]. This study is limited by sample size, but its breadth of professionals across the healthcare field and depth of interview content make it an invaluable resource to understand the reasons behind disparities faced by LEP patients. 

Our study design has many limitations. A response rate of 12.2% is low, which raises significant concerns about nonresponse bias. Students who care about disparities regarding LEP patients may have disproportionately responded to our survey. Additionally, a total student respondent number of 60 is too low for meaningful subgroup analysis. Even the year of schooling, which, when measured against higher self-ratings on training with translation services, would yield useful data about students’ confidence in translated encounters over time, cannot be meaningfully determined with subgroups of less than 20 for each student cohort. Moreover, self-report as a method of data collection is fraught with inaccuracies, personal bias, and even deliberate misreporting in the hopes of achieving a desired outcome. Only 13.3% of students self-reported that they had made assumptions about a patient based upon their presumed culture, ethnicity, or race, but additional students who have made these assumptions may be reluctant to admit it (Table 6). Other limitations include single-institution bias, though this is mitigated somewhat by a multi-site healthcare system with significant variation between clinical sites. That said, a study conducted exclusively with Hackensack Meridian School of Medicine students does limit external validity, considering that a single institutional culture and curriculum structure may not reflect that of other medical schools. Lastly, our study did not include the use of AI-assisted interpretation, which is increasingly common in the United States healthcare system. AI-assisted interpretation is currently not used at a majority of inpatient healthcare sites at the Hackensack Meridian Health network, nor was AI-assisted interpretation used during the year this survey took place. That said, any repeated studies on the Hackensack Meridian School of Medicine student population should assess the percentage of students encountering AI-assisted interpretation and any difficulties they experience.

Our study aimed to provide a descriptive tableau of medical student perceptions of interactions with LEP patients. Our findings indicate multiple areas for future study, including longitudinal student confidence with translation, objective measurements of time spent with LEP patients to accompany our data on student perceptions, and standardized assessments of student cultural competency. However, without quantitative measures of how LEP patients are treated in comparison to English-speaking patients, low-response rate, and a single coder for assignment of qualitative themes, any conclusions must remain tentative at this stage.

## Conclusions

Our study highlights the importance of perceived care gaps in the LEP patient population from a medical student viewpoint. Considering the commonalities between statements regarding LEP patient care perceived by medical students in themselves and in others, our data may point to a set of beliefs that persists throughout subsequent stages of a physician’s career. However, due to the nature of our study as a descriptive cross-sectional survey that relies heavily on self-reporting, our findings should be interpreted as preliminary. 

Future directions should include quantitative measurements of how long healthcare professionals and medical students spend with LEP patients by recording LEP patient encounters. Recordings can be subdivided into time spent acquiring translation materials, time spent setting up those materials, and time spent on the patient interview to determine any rate-limiting steps for further intervention. Quantitative measures can then be correlated with year of schooling or designed as a longitudinal study to follow a single cohort. Additionally, the cross-sectional survey could be repeated in a pre-/post-model alongside a standardized interpreter training. Lastly, assessing students’ cultural competence with a standardized assessment tool, such as the IAPCC-SV, could give insight into root causes for any disparities that are observed.

## Appendices

Appendix A: Survey line items

Demographics

Year in medical school

M1

M2

M3

M4

Gender

Female

Male

Non-binary

Prefer not to say

Race/Ethnicity

American Indian or Alaska Native, non-Hispanic

Asian, non-Hispanic

Black or African American, non-Hispanic

Hispanic

Native Hawaiian or other Pacific Islander, non-Hispanic

White, non-Hispanic 

**Please list any other languages you speak besides English**: _________

Have you ever had to communicate with patients with limited English proficiency (LEP)?

Yes

No 

Select all settings in which you have communicated with patients with LEP

Inpatient

Outpatient

Emergency room

Via telephone

Other 

Select all translation methods you have used with patients

Interpreter via video

Interpreter via phone

In person interpreter

Patient's family member as interpreter

Other healthcare worker as interpreter 

Perspective on using translation services

Do you feel comfortable navigating the use of translation services in a clinical setting?

Yes

No

Did you get training on how to effectively use translator services in a clinical setting?

Yes

No 

**How would you rate your training with translation services?** Poor 1 2 3 4 5 excellent 

If given the choice, what translation method do you prefer to use?

Interpreter via video

Interpreter via phone

In person interpreter

Patient's family member as interpreter

Other healthcare worker as interpreter 

Have you experienced difficulty with any of the following? Select all that apply.

Accessing translator line via telephone

Accessing translator line via video

Finding available iPads for video translation

Finding available in person interpreters

Connecting with an interpreter via phone or video within a timely manner

Need for an interpreter for a rare dialect or language that was not available

Poor internet connection affecting use of translation services

Low volume on phone or video affecting ability to communicate with the patient

Asking open-ended questions while using translation services

Speaking in short sentences while using translation services

Concern for inaccurate translation

None of the above

Medical student perspective on communication with patients with LEP

Do you think there is a difference in how English-speaking patients are cared for by medical students versus patients with LEP?

Yes

No

**Have you experienced any of the following pertaining to patients with LEP? Select all that apply.** 

Felt more hesitancy to see patients with LEP

Perceived seeing patients with LEP as a bigger time commitment

Decreased willingness to revisit/follow up on a patient due to need for obtaining translation

Asking less questions while gathering a history than you would for a patient that does speak English

Asking less open ended questions than you would with a patient who does speak English

Made assumptions about a patient based on their presumed culture, ethnicity, or race without eliciting the patient's beliefs/perspectives

None of the above

What do you find to be the most challenging part of communicating with a patient with LEP? Select all that apply.

Accessing translation services via video

Accessing translation services via phone

Accessing translation services via in person interpreter

Phrasing questions in a way that is easy to be translated

Accounting for possible increased time spent with patient due to need for translation use

Forming a connection with a patient while using translation services

Expressing empathy to the patient

Conducting a physical exam while using interpreter

Other 

Observation about other providers 

Do you think there is a difference in how members of the healthcare team treat patients who do not speak English compared to patients with LEP?

Yes

No 

Have you witnessed any of the following by other healthcare providers in relation to patients with LEP? Select all that apply.

Longer wait times for patients to be seen by healthcare providers

Decreased number of visits/follow up from provider while patient is hospitalized

Dismissal of patient complaints or concerns

Assumptions about a patient based on their presumed culture, ethnicity, or race without actually eliciting the patient's belief/perspective

More readily labeled "noncompliant"

No use of translation services for patients with LEP

Less time spent interviewing patients

Less time spent explaining diagnosis or next steps

Less time spent answering patient questions

Less open ended questions asked

Different treatment/testing options offered to patients with LEP compared to those who do

None of the above

Possible solutions 

Do you think any of the following would improve medical student interactions with patients with LEP? Select all that apply.

Improved training on how to use translation services effectively

Refresher training on use of translation services directly before beginning clerkships.

Training on how to use translation services specifically in a clinical setting

More information provided on what translation services are available at each clinical site

Improved identification of patients with LEP in Epic

Timer added to Epic to indicate how long patients with LEP have been waiting to be seen by a provider

Increased access to translation services (i.e., more iPads available)

Decreased wait times for available translators (i.e., more translators available)

Other
